# RAE1 is a prognostic biomarker and is correlated with clinicopathological characteristics of patients with hepatocellular carcinoma

**DOI:** 10.1186/s12859-022-04806-8

**Published:** 2022-06-24

**Authors:** Gang Chi, Jin-Hong Pei, Xue-Qing Li

**Affiliations:** Department of Biochemistry, Changzhi Medical College, Changzhi, 046000 Shanxi China

**Keywords:** RAE1, Hepatocellular carcinoma, Prognosis, Microtubules

## Abstract

**Background:**

Hepatocellular carcinoma (HCC) is a primary malignant tumor that accounts for approximately 90% of all cases of primary liver cancer worldwide. Microtubule alterations may contribute to the broad spectrum of resistance to chemotherapy, tumor development, and cell survival. This study aimed to assess the value of ribonucleic acid export 1 (RAE1), as a regulator of microtubules, in the diagnosis and prognosis of HCC, and to analyze its correlation with genetic mutations and pathways in HCC.

**Results:**

The mRNA and protein levels of RAE1 were significantly elevated in HCC tissues compared with those in normal tissues. The high expression level of RAE1 was correlated with T stage, pathologic stage, tumor status, histologic grade, and alpha-fetoprotein level. HCC patients with a higher expression level of RAE1 had a poorer prognosis, and the expression level of RAE1 showed the ability to accurately distinguish tumor tissues from normal tissues (area under the curve (AUC) = 0.951). The AUC values of 1-, 3-, and 5-year survival rates were all above 0.6. The multivariate Cox regression analysis showed that RAE1 expression level was an independent prognostic factor for a shorter overall survival of HCC patients. The rate of RAE1 genetic alterations was 1.1% in HCC samples. Gene ontology and kyoto encyclopedia of genes and genomes pathway enrichment analyses indicated the co-expressed genes of RAE1 were mainly related to chromosome segregation, DNA replication, and cell cycle checkpoint. Protein–protein interaction analysis showed that RAE1 was closely correlated with NUP205, NUP155, NUP214, NUP54, and NXF1, all playing important roles in cell division and mitotic checkpoint.

**Conclusion:**

RAE1 can be a potential diagnostic and prognostic biomarker associated with microtubules and a therapeutic target for HCC.

**Supplementary Information:**

The online version contains supplementary material available at 10.1186/s12859-022-04806-8.

## Background

Hepatocellular carcinoma (HCC), as a major type of primary liver cancer, accounts for more than 80% of malignant primary liver tumors [1–3]. The incidence of HCC is still increasing annually and ranks sixth among the most common malignant tumors, making it the second most leading cause of cancer-related death worldwide [4, 5]. The majority of HCC patients are mainly diagnosed at the advanced-stage with a poor prognosis due to its asymptomatic development [6, 7]. At present, although surgical resection and liver transplantation are potential curative therapies for early-stage HCC, treatment with small-molecule multi-kinase inhibitors (e.g., sorafenib and regorafenib) is the first-line therapy for patients with advanced-stage HCC [8–10]. However, these drugs have still very low therapeutic rates for HCC and are limited by primary and acquired resistance [11, 12]. Thus, it is urgent to explore novel biomarkers and effective therapeutic targets for identifying high-risk patients with HCC, in order to develop more effective therapeutic strategies.

Microtubules are essential components of the cytoskeleton, and they are an important molecular target for novel therapeutics in cancer [13]. Microtubules are involved in a variety of biological processes, including cell division, apoptosis, proliferation, and angiogenesis, because they constitute the mitotic spindle, which is a complex structure that coordinates the accurate segregation of chromosomes during cell division. The dynamic characteristics of depolymerization and polymerization of spindle microtubules are required for cells to complete mitosis. Impairment in the dynamic behavior would affect the division of tumor cells and inhibit tumor growth [14–16]. Thus, it is essential to determine these key molecules that regulate microtubule assembly and function.

Ribonucleic acid export 1 (RAE1) has a microtubule–associated activity and influences microtubule functions in higher eukaryotes. RAE1 binds to microtubules and is required for formation of a mitotic spindle [17, 18]. RAE1 overexpression was detected in breast cancer cells, which could promote proliferation of human cancer cells by the Hippo signaling pathway. Moreover, RAE1 was found to be positively correlated with gene copy number, which promoted a more aggressive phenotype and induced epithelial-mesenchymal transition (EMT). Furthermore, RAE1 overexpression was positively correlated with the histologic grade and a poor prognosis in patients with breast cancer and colorectal cancer [13, 19–22]. However, the detailed mechanisms of RAE1 in HCC have still remained elusive.

In the present study, we comprehensively analyzed RAE1 expression level and its correlation with prognosis of HCC patients. The results revealed the significant prognostic value and potential role of RAE1, which may assist scholars to better understand the association between RAE1 expression level and prognosis of HCC patients.

## Methods

### TIMER database analysis

Tumor immune estimation resource (TIMER) is a comprehensive database that analyzes immune infiltration in diverse types of cancer [23]. We used TIMER to evaluate the differences in expression level of RAE1 between tumor and adjacent normal tissues at the transcription level of various types of cancer. The “correlation” module in the TIMER database was used to analyze the correlation between RAE1 expression level and the common genes from intersection analysis. *P* < 0.05 was considered statistically significant.

### UALCAN database analysis

The UALCAN database is an interactive web portal to perform in-depth analyses of gene expression and clinical data from The Cancer Genome Atlas (TCGA) database [24]. In the present study, the UALCAN database was used to explore the expression levels of RAE1 mRNA and protein in primary HCC tissues and their association with clinicopathological characteristics.

### Kaplan–Meier plotter database analysis

The Kaplan–Meier plotter is an online database, containing survival data of 21 types of cancer [25]. In the present study, the relationship between RAE1 expression level and overall survival (OS), progression-free survival (PFS), disease-specific survival (DSS), and relapse-free survival (RFS) was assessed by the Kaplan–Meier survival analysis. Hazard ratios (HRs) with 95% confidence intervals (CIs) and log-rank P values were also calculated and presented on the graphs.

### Diagnostic analysis

Receiver operating characteristic (ROC) curves, time-dependent curves, and a nomogram model were created for diagnosis using R packages (pROC, timeROC, rms, ggplot2, and survival packages). The clinical data were retrieved from TCGA database.

### cBioPortal database analysis

The cBioPortal is an online database that was developed to explore, visualize, and analyze multidimensional cancer genomics data [26]. We used the cBioPortal to analyze the genomic profiles of RAE1 in HCC using three datasets (TCGA, Firehose Legacy; INSERM, Nat genet 2015 and AMC Hepatology 2014). Kaplan–Meier plots were drawn and the log-rank test was used to identify the significant differences between the survival curves, and significant differences were statistically defined as *P* < 0.05.

### LinkedOmics database analysis

The LinkedOmics database is a public platform, providing comprehensive multi-omics data from 11,158 patients for 32 types of cancer [27]. In the current study, RAE1-related differentially expressed genes (DEGs) were screened in HCC samples using the “LinkFinder” module. The correlation results were tested by the Pearson correlation analysis and visualized via volcano and heat maps. The RAE1-related DEGs were annotated using Gene Ontology biological process (GO_BP) in the “Function” module. The Kyoto Encyclopedia of Genes and Genomes (KEGG) pathway was analyzed by the gene set enrichment analysis (GSEA) using the “LinkInterpreter” module.

### Protein–protein interaction (PPI) analysis

The STRING database was used to investigate the PPI network information of RAE1. The interaction was considered statistically significant if protein interaction score was > 0.9. The interactive Venn diagram was used to perform the intersection analysis to compare significantly associated genes of RAE1 and interacted genes of RAE1.

### Statistical analysis

The difference in RAE1 expression level between tumor tissues and normal tissues was compared using the Wilcoxon test. RAE1 expression level in different Clinicopathologic characteristics and HR and 95% CI in survival analysis were evaluated by univariate Cox regression. The survival analysis was performed using Kaplan–Meier curves. Multivariate Cox regression analysis was used to compare the effects of RAE1 expression level on survival rate along with other clinicopathological characteristics. The statistical analysis was performed using R 4.1.2 software, and *P* < 0.05 was considered statistically significant.

### The statement

We confirm that all methods were carried out in accordance with relevant guidelines and regulations or declaration of Helsinki.

## Results

### Differential expression of RAE1 in pan-cancer and HCC

**Fig. 1:**
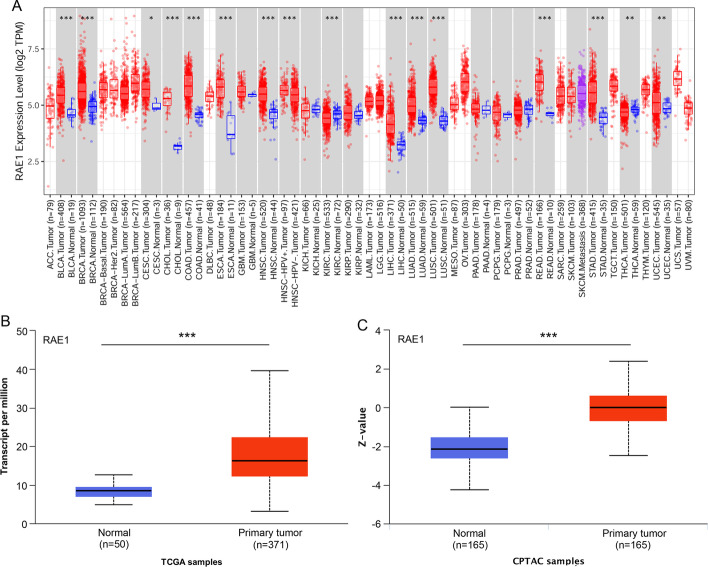
The expression level of RAE1 in pan-cancer and HCC. **A** RAE1 expression level in various types of cancer was analyzed using Tumor Immune Estimation Resource (TIMER) database. **B** The expression level of RAE1 mRNA was determined in normal and HCC tissues using TCGA samples by the UALCAN database analysis. **C** The expression level of RAE1 protein in normal and HCC tissues was analyzed using CPTAC samples by the UALCAN database analysis. *P < 0.05, **P < 0.01, ***P < 0.001

To investigate the difference in RAE1 expression level in various types of human cancer, the TIMER database was used to analyze RAE1 expression level in normal and tumor tissues. Compared with normal tissues, RAE1 expression level was significantly upregulated in tissues of bladder urothelial carcinoma (BLCA), breast invasive carcinoma (BRCA), cervical squamous cell carcinoma and endocervical adenocarcinoma (CESC), cholangiocarcinoma (CHOL), colon adenocarcinoma (COAD), esophageal carcinoma (ESCA), head and neck squamous cell carcinoma (HNSC), liver hepatocellular carcinoma (LIHC), lung adenocarcinoma (LUAD), lung squamous cell carcinoma (LUSC), rectal adenocarcinoma (READ), stomach adenocarcinoma (STAD), and uterine corpus endometrial carcinoma (UCEC). However, RAE1 expression level was significantly downregulated in tissues of kidney renal clear cell carcinoma (KIRC) and thyroid carcinoma (THCA) (Fig. 1A). To further determine RAE1 expression level in LIHC, the UALCAN database was used to analyze the expression levels of RAE1 mRNA and protein in normal and tumor tissues. The expression levels of RAE1 mRNA and protein were significantly elevated in HCC tissues (Fig. 1B, C). These results suggested that the expression levels of RAE1 mRNA and protein were significantly upregulated in HCC tissues.

### Relationship between RAE1 expression level and clinicopathological characteristics of HCC patients

**Fig. 2:**
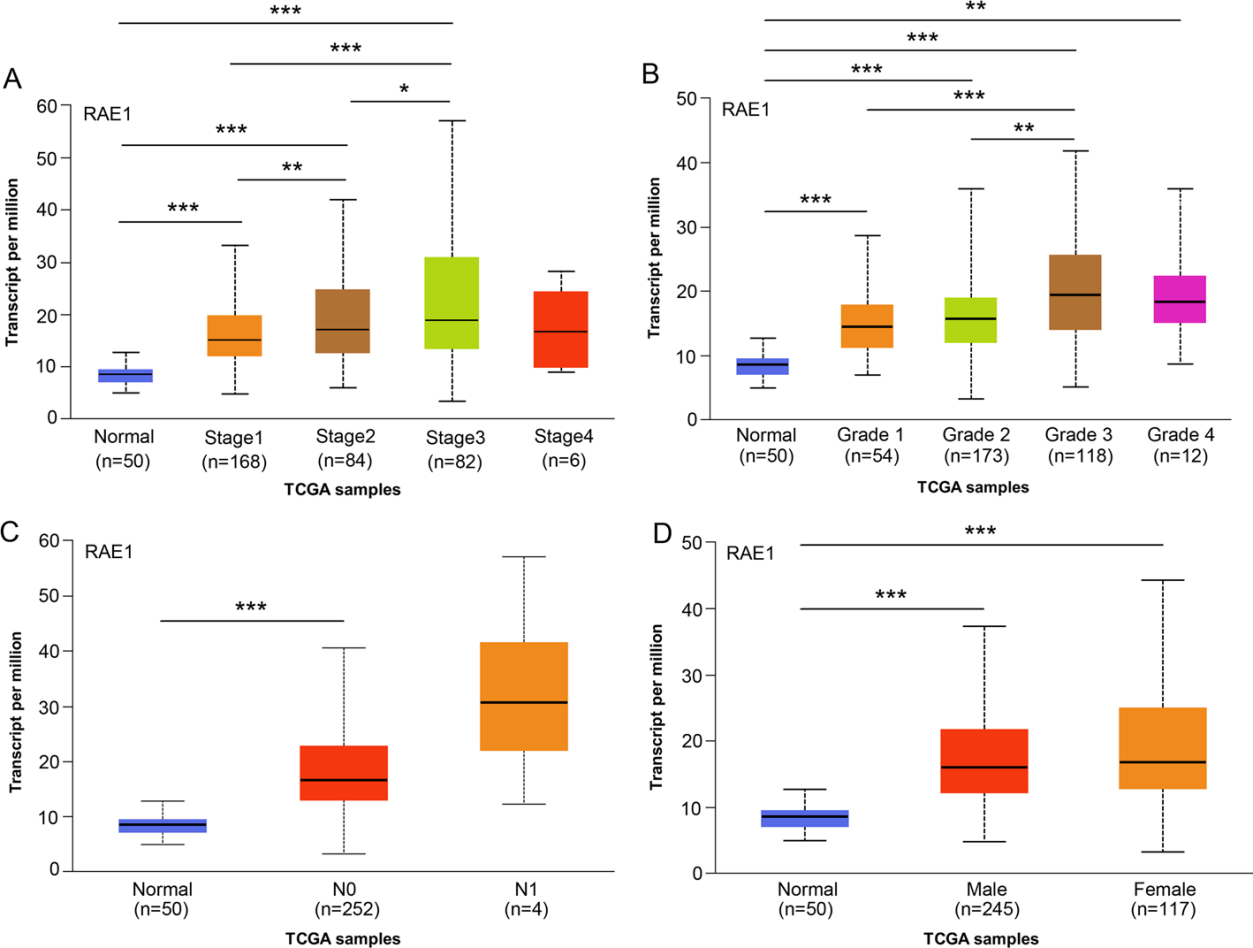
Association of RAE1 mRNA expression level with clinicopathological characteristics of patients with HCC. **A** The expression level of RAE1 mRNA was significantly correlated with pathologic stage in HCC samples from the UALCAN database. **B** The expression level of RAE1 mRNA was correlated with histologic grade from the UALCAN database. **C** The expression level of RAE1 mRNA was correlated with N stage from the UALCAN database. **D** The expression level of RAE1 mRNA was correlated with gender (male and female) from the UALCAN database

To determine the correlation of RAE1 expression level with clinicopathological characteristics of HCC patients, TCGA database was used. The high expression level of RAE1 was significantly correlated with T stage (*P* < 0.001), pathologic stage (*P* < 0.001), tumor status (*P* = 0.012), histologic grade (*P* < 0.001), and alpha-fetoprotein (AFP) level (*P* < 0.001) (Additional file 1 and Table 1). The UALCAN database was utilized to determine the relationship between RAE1 mRNA expression level and clinicopathological characteristics. The highest expression level of RAE1 was found in stage 3, grade 3, N0, and female (Fig. 2). As shown in Table 2, univariate logistic regressions revealed that RAE1 expression level, as a categorical dependent variable, was significantly associated with T stage (T2&T3&T4 vs. T1, odds ratio (OR) = 2.03, *P* < 0.001), pathologic stage (Stage III vs. Stage I, OR = 2.444, *P* = 0.001), tumor status (with tumor vs. tumor-free, OR = 1.801, *P* = 0.007), histologic grade (G3 vs. G1, OR = 2.543, *P* = 0.005), and AFP level (> 400 vs. <  = 400, OR = 2.663, *P* < 0.001). These results showed that a high expression level of RAE1 was significantly associated with clinicopathological characteristics of HCC patients.

**Table 1:** Association between RAE1 expression and clinical features in HCC patients

Characteristic	Low expression of RAE1	High expression of RAE1	*P*
Age			0.435
< = 60	93 (24.9%)	84 (22.5%)	
> 60	94 (25.2%)	102 (27.3%)	
Gender			0.269
Female	55 (14.7%)	66 (17.6%)	
Male	132 (35.3%)	121 (32.4%)	
T stage			** < 0.001**
T1	110 (29.6%)	73 (19.7%)	
T2	43 (11.6%)	52 (14%)	
T3	28 (7.5%)	52 (14%)	
T4	3 (0.8%)	10 (2.7%)	
N stage			0.623
N0	124 (48.1%)	130 (50.4%)	
N1	1 (0.4%)	3 (1.2%)	
Pathologic stage			** < 0.001**
Stage I	103 (29.4%)	70 (20%)	
Stage II	41 (11.7%)	46 (13.1%)	
Stage III	29 (8.3%)	56 (16%)	
Stage IV	3 (0.9%)	2 (0.6%)	
Tumor status			**0.012**
Tumor free	113 (31.8%)	89 (25.1%)	
With tumor	64 (18%)	89 (25.1%)	
Histologic grade			** < 0.001**
G1	35 (9.5%)	20 (5.4%)	
G2	101 (27.4%)	77 (20.9%)	
G3	45 (12.2%)	79 (21.4%)	
G4	4 (1.1%)	8 (2.2%)	
AFP(ng/ml)			** < 0.001**
< = 400	125 (44.6%)	90 (32.1%)	
> 400	21 (7.5%)	44 (15.7%)	
Vascular invasion			0.165
No	113 (35.5%)	95 (29.9%)	
Yes	50 (15.7%)	60 (18.9%)	

Black fonts indicate *P* < 0.05

**Table 2:** Logistic regression analysis of the association between RAE1 expression and clinicopathological characteristics in HCC patients

Characteristics	Total (N)	Odds ratio (OR)	*P* value
T stage (T2&T3&T4 vs. T1)	371	2.030 (1.345–3.078)	** < 0.001**
N stage (N1 vs. N0)	258	2.729 (0.344–55.579)	0.387
M stage (M1 vs. M0)	272	0.971 (0.115–8.185)	0.976
Pathologic stage (Stage III vs. Stage I)	258	2.444 (1.439–4.207)	**0.001**
Age (> 60 vs. < = 60)	373	0.928 (0.618–1.394)	0.719
Gender (male vs. female)	374	0.802 (0.519–1.238)	0.320
Tumor status (with tumor vs. Tumor free)	355	1.801 (1.180–2.763)	**0.007**
Histologic grade (G3 vs. G1)	179	2.543 (1.335–4.929)	**0.005**
Vascular invasion (Yes vs. No)	318	1.376 (0.866–2.192)	0.177
AFP(ng/ml) (> 400 vs. < = 400)	280	2.663 (1.504–4.826)	** < 0.001**

Black fonts indicate *P* < 0.05

### Prognostic value of RAE1 expression level in HCC patients

**Fig. 3:**
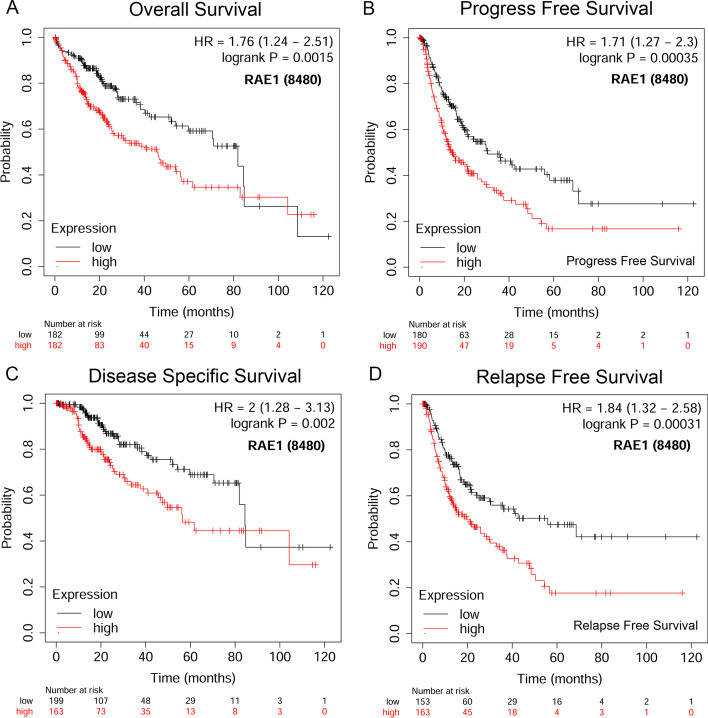
The prognostic analysis of RAE1 expression level in HCC patients (Kaplan–Meier plotter). The survival curves revealed that a higher RAE1 expression level was significantly associated with a shorter OS (**A**), a shorter PFS (**B**), a shorter DSS (**C**), and a shorter RFS (**D**)

**Fig. 4:**
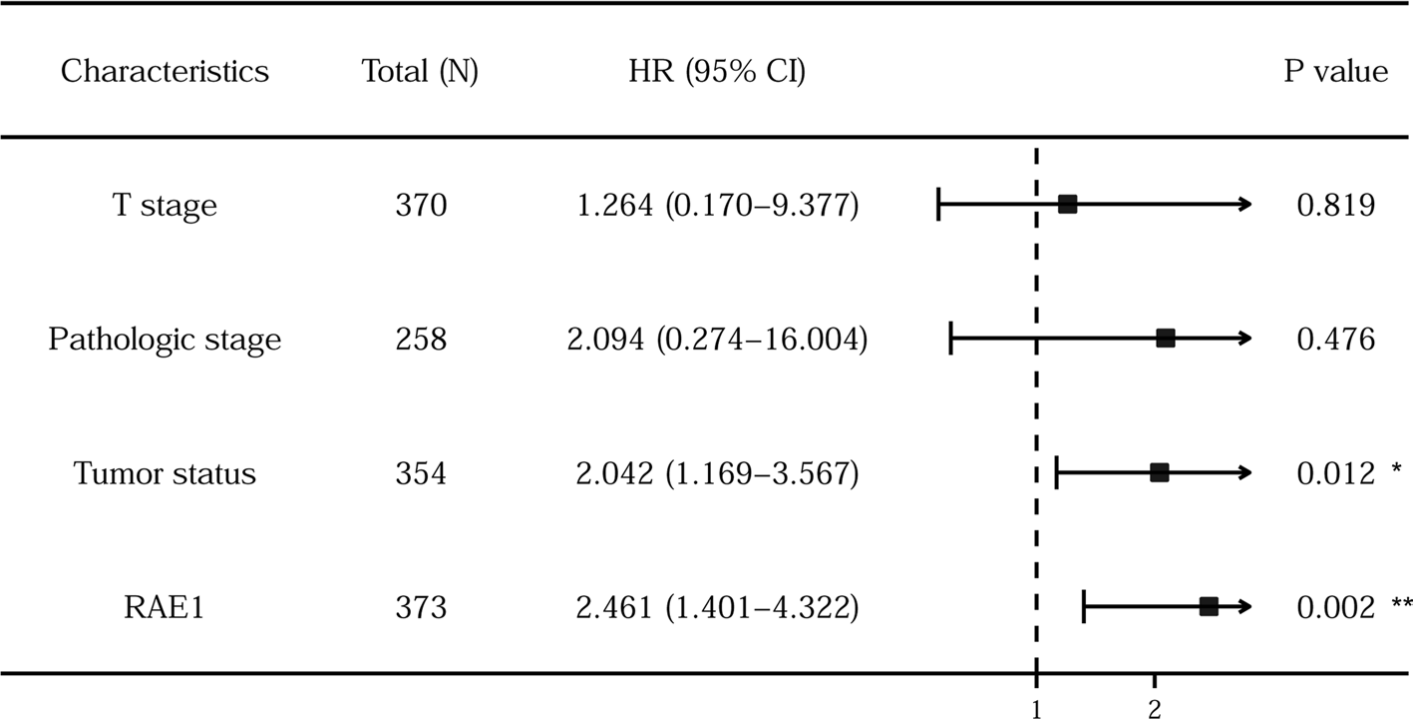
Multivariate Cox regression analysis of RAE1 expression level and other clinicopathological characteristics. The 95% CIs for the HRs and associated P-values were displayed in box. HR greater than 1 and *P* < 0.05 indicate that a high gene expression or clinical pathological factors are associated with a poor overall survival of HCC patients. CI, confidence interval; HR, hazard ratio

It was attempted to indicate whether RAE1 expression level could affect prognostic value of HCC patients. The Kaplan–Meier plotter was used to analyze the prognostic value of RAE1 expression level in HCC patients. The results showed that a higher expression level of RAE1 was significantly associated with a poor OS (HR = 1.76, *P* = 0.0015), a poor PFS (HR = 1.71, *P* = 0.00035), a poor DSS (HR = 2, *P* = 0.002), and a poor RFS (HR = 1.84, *P* = 0.00031) in HCC patients (Fig. 3). Univariate Cox regression analysis indicated that T stage, M stage, pathologic stage, tumor status, and RAE1 expression level were significantly correlated with a poor OS. Moreover, the multivariate Cox regression analysis showed that tumor status (HR = 2.042, *P* = 0.012) and RAE1 expression level (HR = 2.461, *P* = 0.002) were independent prognostic factors of OS in HCC patients (Table 3 and Fig. 4). These results suggested that a high expression level of RAE1 was associated with a worse prognosis.

**Table 3:** Cox regression analysis between prognostic risk factors and overall survival in patients with HCC

Risk factors	Univariate analysis		Multivariate analysis	
	Hazard ratio (95% CI)	*P* value	Hazard ratio (95% CI)	*P* value
Age	1.205 (0.850–1.708)	0.295		
Gender	0.793 (0.557–1.130)	0.200		
T stage	2.126 (1.481–3.052)	** < 0.001**	1.264 (0.170–9.377)	0.819
N stage	2.029 (0.497–8.281)	0.324		
M stage	4.077 (1.281–12.973)	**0.017**		
Pathologic stage	2.724 (1.783–4.159)	** < 0.001**	2.094 (0.274–16.004)	0.476
Histologic grade	1.178 (0.678–2.045)	0.561		
Tumor status	2.317 (1.590–3.376)	** < 0.001**	2.042 (1.169–3.567)	**0.012**
Vascular invasion	1.344 (0.887–2.035)	0.163		
AFP(ng/ml)	1.075 (0.658–1.759)	0.772		
RAE1	1.941 (1.364–2.763)	** < 0.001**	2.461 (1.401–4.322)	**0.002**

Black fonts indicate *P* < 0.05

### Diagnostic and prognostic values of RAE1 expression level in HCC patients

**Fig. 5:**
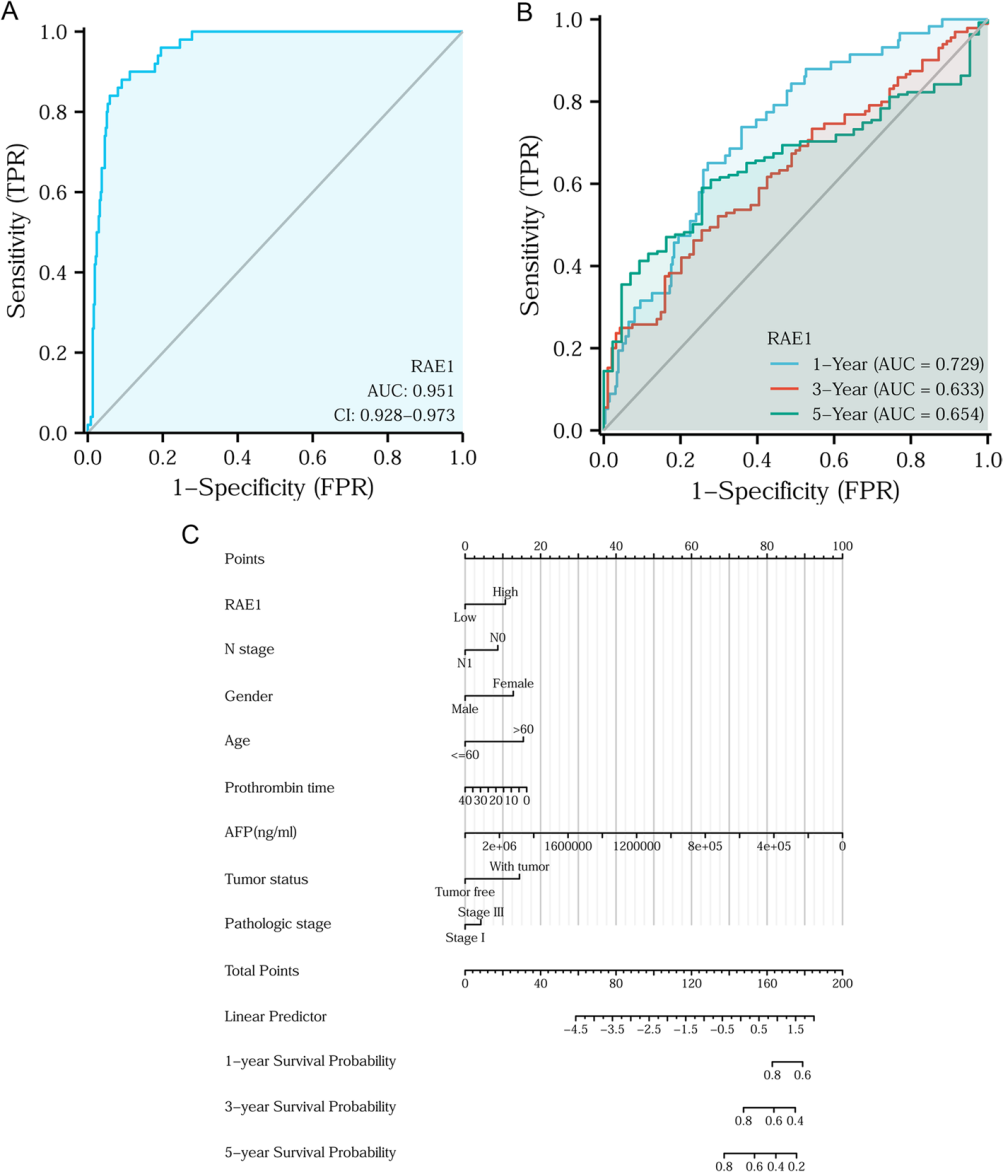
ROC curves and a nomogram model for predicting diagnostic and prognostic values of RAE1 expression level. **A** The ROC curve was used to identify tumor tissue from normal tissue. **B** Time-dependent survival ROC curve was used to predict 1-, 3-, and 5-year survival rates. **C** Nomogram model was used to predict survival probability at 1-, 3-, and 5-year through integrating clinicopathological characteristics and RAE1 expression level

To further evaluate the diagnostic value of RAE1 expression level, the ROC curve analysis was performed to assess the diagnostic accuracy of RAE1. The expression level of RAE1 showed the ability to accurately distinguish tumor tissues from normal tissues (area under the curve (AUC) = 0.951) (Fig. 5A). Time-dependent ROC curve of RAE1 expression level was used to evaluate 1-, 3-, and 5-year survival rates. All the AUC values were considered appropriate for prediction (AUC > 0.6) (Fig. 5B). A nomogram was constructed to predict the survival probability at 1-, 3-, and 5-year by integrating RAE1 expression level and clinicopathological characteristics (N stage, gender (male or female), age, prothrombin time (PT), AFP level, tumor status, and pathologic stage) (Fig. 5C).

### Genetic alterations of RAE1 in HCC patients

**Fig. 6:**
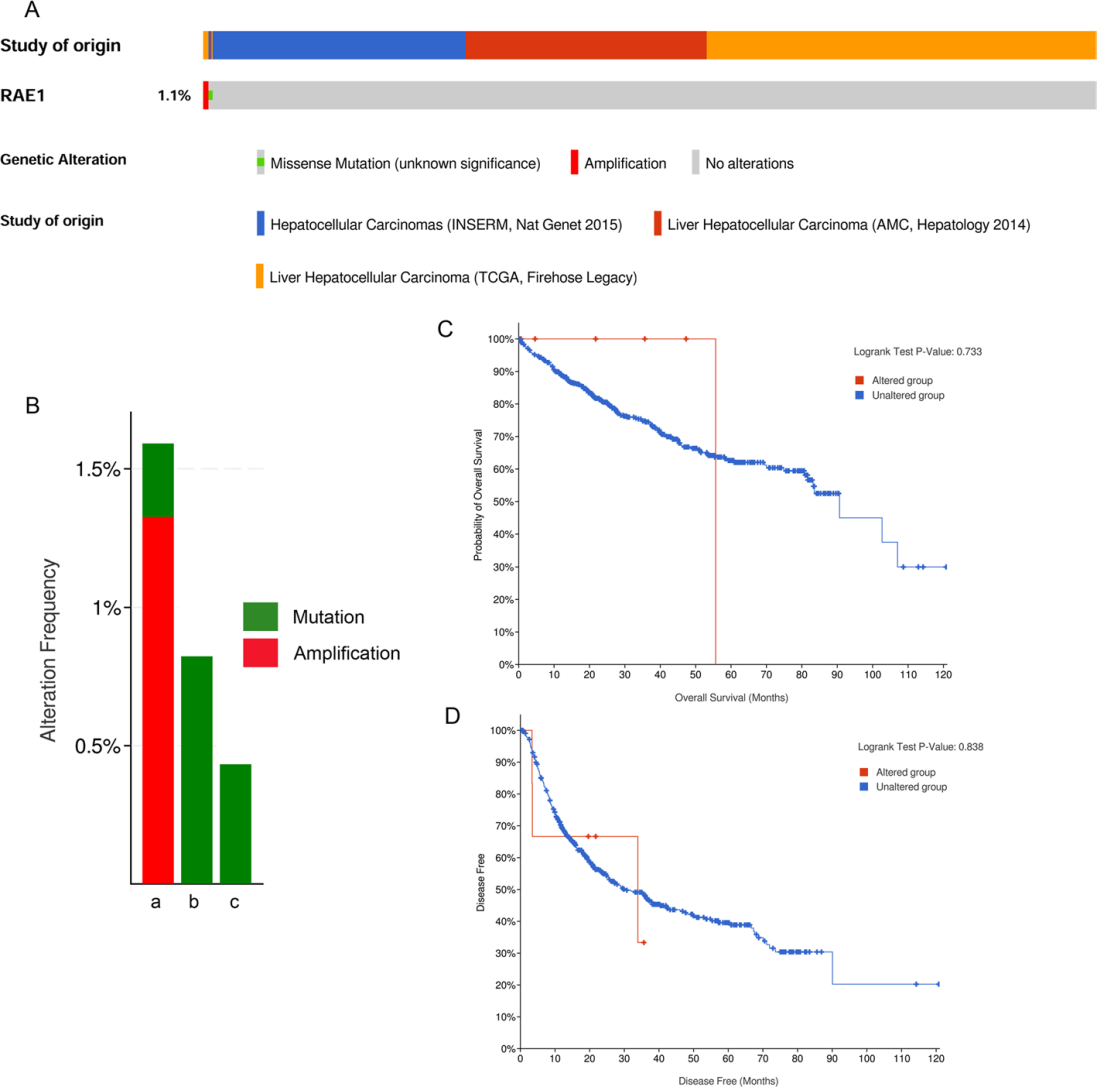
Genetic alterations of RAE1 and their association with OS and DFS in HCC patients. **A** OncoPrint visualization of genetic alterations of RAE1. **B** Summary of genetic alterations of RAE1 in HCC samples from TCGA, Firehose Legacy (a); INSERM, Nat genet 2015 (b) and AMC Hepatology 2014 (c). Kaplan–Meier plots comparing overall survival **C** and disease-free survival **D** in patients with/without genetic alterations of RAE1

Next, we analyzed genetic alterations of RAE1 using three datasets (TCGA, Firehose Legacy; AMC, Hepatology 2014; INSERM, Nat Genet 2015) from the cBioPortal online platform. RAE1 was altered in 9 of 851 (1.1%) HCC patients (Fig. 6A). The alteration rate ranged from 0.43 (1/231) to 1.59% (6/377) (Fig. 6B). Furthermore, Kaplan–Meier plots and the log-rank test showed that these genetic alterations caused no significant difference in OS (*P* = 0.733) and DFS (*P* = 0.838) in HCC patients (Fig. 6C, D).

### Co-expression pattern of RAE1 in HCC

**Fig. 7:**
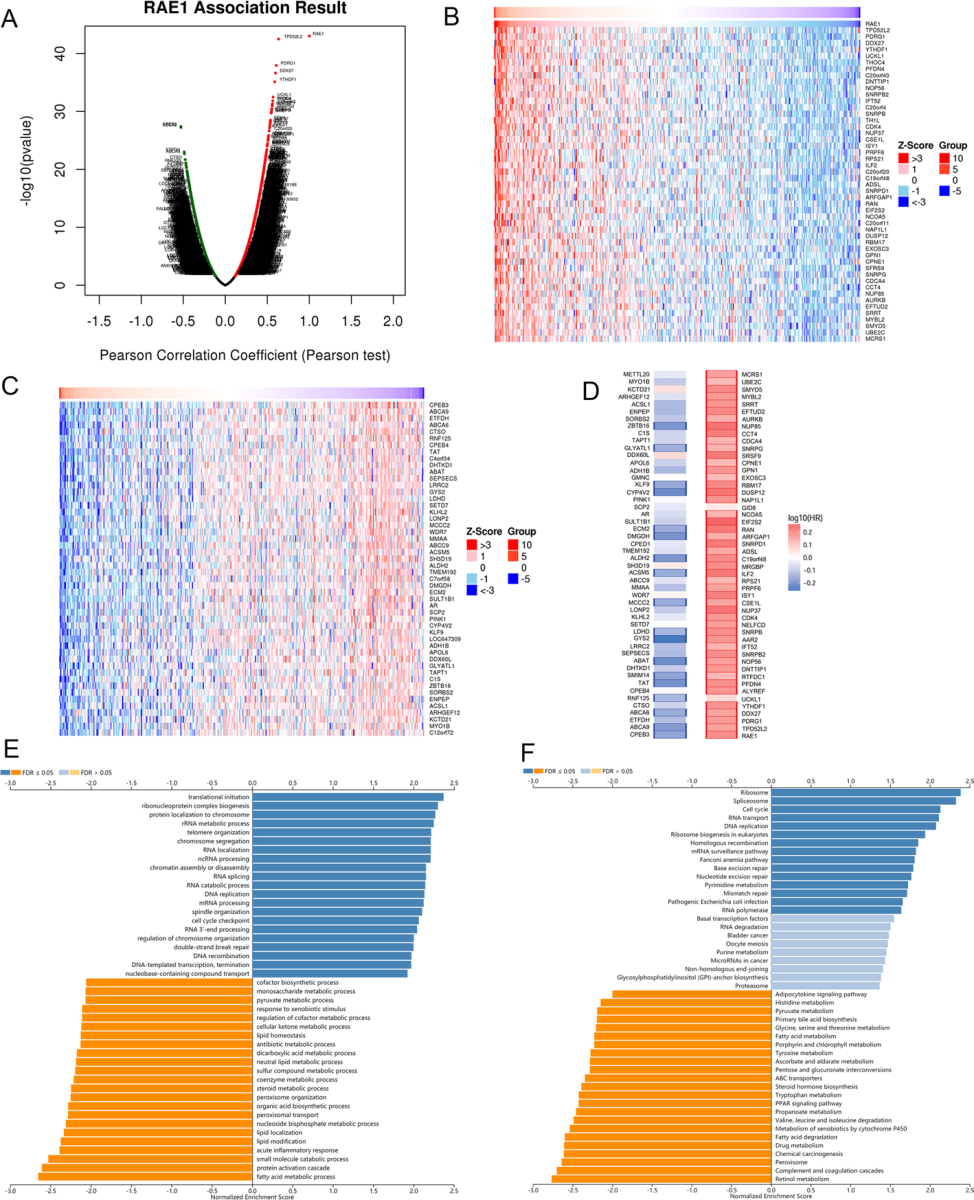
The co-expression network analysis of RAE1 in HCC samples from the LinkedOmics database. **A** The genes significantly associated with RAE1 in HCC samples could be distinguished by the LinkedOmics database. (**B** and **C**) Top 50 genes positively (**B**) and negatively (**C**) associated with RAE1 in HCC are displayed by the heat map. **D** Survival map of the top 50 genes positively and negatively related to RAE1 in HCC. (**E** and **F**) RAE1 co-expression genes in HCC were annotated by GO annotations **E** and KEGG pathways (www.kegg.jp/kegg/kegg1.html) **F** in the LinkedOmics

To further explore biological function of RAE1 in HCC, the “LinkFinder” module of LinkedOmics database was used to analyze the co-expression pattern of RAE1 in HCC. The results showed that 6295 genes were positively correlated with RAE1, while 3608 genes were negatively correlated with RAE1 (Fig. 7A). The top 50 genes, which were positively and negatively associated with RAE1, were presented in heat maps (Fig. 7B, C). It is noteworthy that 48 genes represented unfavorable HRs among the top 50 positively significant genes, which become high-risk markers. Comparably, 41 genes had protective HRs in the top 50 negatively significant genes (Fig. 7D). The annotation of GO terms showed that co-expressed genes of RAE1 were mainly enriched in translational initiation, ribonucleoprotein complex biogenesis, protein localization to chromosome, rRNA metabolic process, telomere organization, chromosome segregation, DNA replication and cell cycle checkpoint, etc. (Fig. 7E). The KEGG pathway enrichment analysis indicated enrichment in ribosome, cell cycle, RNA transport, DNA replication, base excision repair, mismatch repair, mRNA surveillance pathway, etc. (Fig. 7F). These results revealed that RAE1 expression pattern influenced the DNA replication and repair, RNA transcription and translation, and cell cycle process in HCC.

### PPI network analysis of RAE1-related genes

**Fig. 8:**
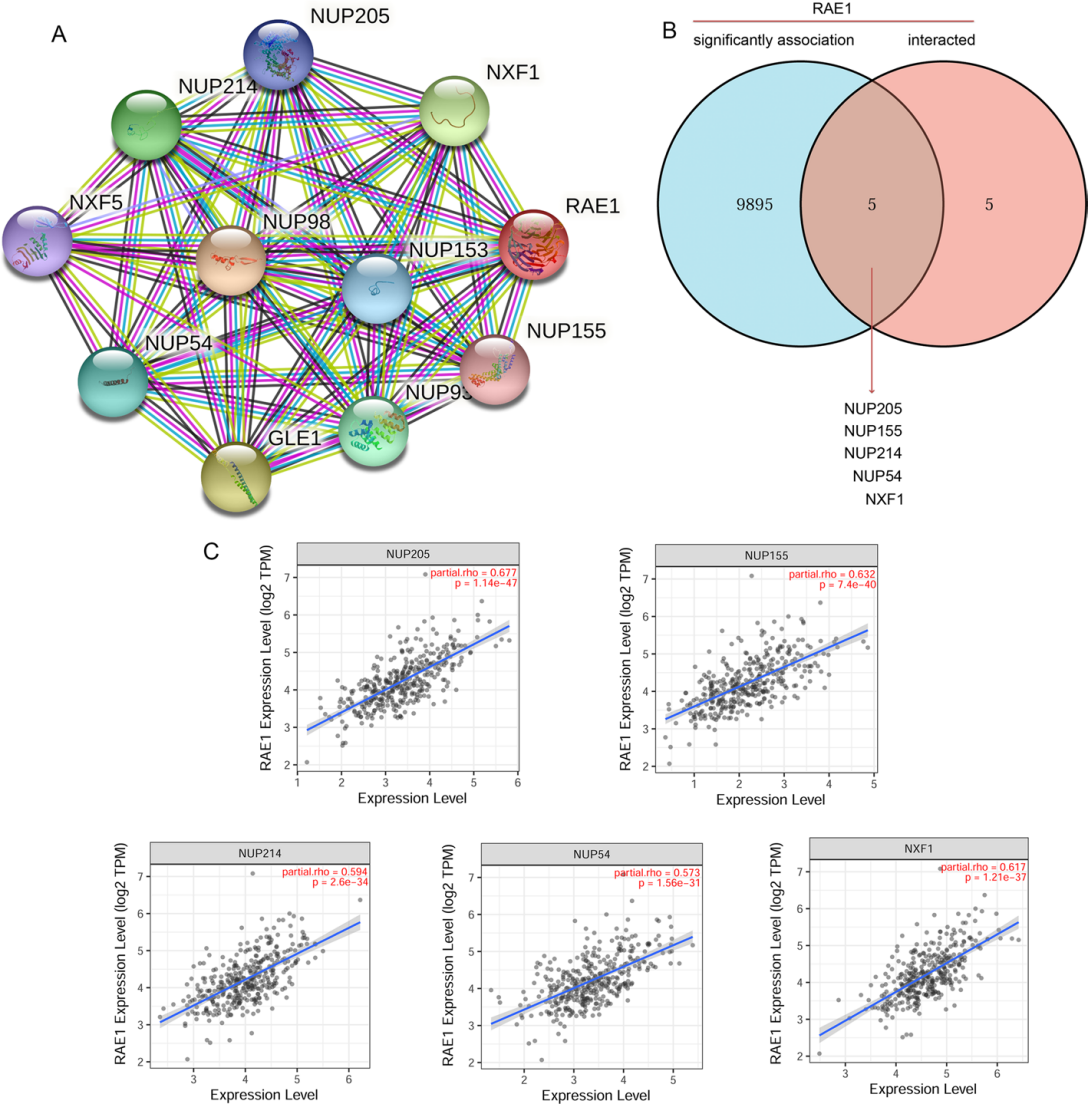
PPI network analysis of RAE1 related genes. **A** The visualizing network of RAE1-interacted proteins based on STRING database. **B** An intersection point between RAE1-significantly associated genes and RAE1-interacted genes was analyzed. **C** Correlation analysis of RAE1 expression level with screened common genes, including NUP205, NUP155, NUP214, NUP54, and NXF1

To further explore the possible molecular mechanism of RAE1 gene in HCC tumorigenesis, the PPI network analysis was performed using the STRING online database. The top 10 functional partner proteins related to RAE1 were selected and visualized (Fig. 8A). In addition, a total of 5 common members (NUP205, NUP155, NUP214, NUP54, and NXF1) were screened through comparing significantly associated genes of RAE1 with interacted genes of RAE1 (Fig. 8B). Moreover, RAE1 expression level was significantly positively correlated with NUP205 (r = 0.677, *P* = 1.14e−47), NUP155 (r = 0.632, *P* = 7.4e−40), NUP214 (r = 0.594, *P* = 2.6e−34), NUP54 (r = 0.573, *P* = 1.56e−31), and NXF1 (r = 0.617, *P* = 1.21−37) (Fig. 8C).

## Discussion

RAE1, a mitotic checkpoint regulator, is one of the 30 different proteins found in the nuclear pore complex (NPC). The overexpression and dysregulation of RAE1 could lead to chromosome missegregation, instability and multipolar spindles, contributing to cancer development and progression [19, 21]. RAE1 downregulation reduced the migration and invasion capabilities of breast cancer cells. RAE1 overexpression could facilitate EMT to lead to an invasive ductal histology and a high histological grade in breast cancer. RAE1 also promoted colorectal tumor growth through inhibiting apoptosis and promoting cell cycle progression in part by stabilization of spindle bipolarity [13, 19]. To date, the role of RAE1 in HCC has not been investigated.

In the present study, we analyzed RAE1 expression profile in different types of human cancer using the TIMER database. The results indicated that compared with normal tissues, RAE1 expression level was higher in tissues of BLCA, BRCA, CESC, CHOL, COAD, ESCA, HNSC, LIHC, LUAD, LUSC, READ, STAD, and UCEC, whereas RAE1 expression level was lower in tissues of KIRC and THCA. We also examined the mRNA and protein expression levels of RAE1 in HCC and normal tissues using the UALCAN database, and the results showed that mRNA and protein expression levels of RAE1 increased in HCC tissues. An elevated RAE1 expression level was correlated with a higher T stage, pathologic stage, tumor status, histologic grade, and AFP level. A high expression level of RAE1 was also significantly associated with distant metastasis [13]. Importantly, RAE1 expression level was correlated with AFP level in the present study. AFP is the approved marker for screening HCC among serological biomarkers, while it has an insufficient sensitivity to detect early-stage HCC patients, because AFP level is correlated with the extent of tumor burden. The GALAD score (gender (G), age (A), AFP-L3 (L), AFP (A), and DCP (D)) was successfully tested for detection of early and non-cirrhotic HCC patients to address the limitations of AFP in the detection of early-stage HCC patients, although RAE1 expression level was found independent of age (A) and gender (G) in the present study. GALAD could detect HCC independent of cirrhosis or stage of HCC [28, 29]. The serological approach for detection of early-stage HCC patients is very simple and rapid, as it is operator-independent and the GALAD score could easily be implemented in laboratory information management systems [28]. Thus, the efficiency and sensitivity of the early-stage detection of HCC patients may be improved using the combination of GALAD score and RAE1 expression level. In addition to AFP, the key molecular markers in the serum correlated with RAE1 expression level should be explored in the future study to accurately diagnose early-stage HCC patients and to identify potential targets for early treatment.

In the present study, the Kaplan–Meier plotter was used to analyze the prognostic value of RAE1 expression level. A high expression level of RAE1 was significantly associated with shorter OS, PFS, DSS, and RFS. Multivariate Cox regression analysis showed that RAE1 expression level was an independent prognostic factor for a shorter OS of HCC patients. The OS is an ideal primary endpoint for patients, as it encompasses the potential mortality arising from the tumor itself or treatment or other causes [30]. However, it has been shown that OS has the disadvantage of requiring a long-term follow-up and it can be influenced by subsequent treatment lines, especially in the adjuvant treatment. The main endpoint used to define the efficacy of adjuvant therapy was RFS [31]. RFS could potentially expedite approval of a new drug, and it could not be influenced by subsequent treatments. PFS is an important factor in tumor survival analysis, and numerous clinical trials have reported significant differences in PFS, rather than in OS. PFS is a more sensitive endpoint for treatment effect, and it is also independent of post-progression survival [32]. However, PFS is very difficult to be accurately determined using patients’ records because it is generally confirmed by monitoring, and patients may be progressed prior to performing scanning [33]. The analysis of DSS trend is of great significance to determine whether patient outcomes change over time, and it is also advantageous to evaluate the effects of new interventions on mortality rate [34]. Importantly, a high expression level of RAE1 resulted in poor OS, PFS, RFS, and DSS, indicating that a high expression level of RAE1 was associated with the reduced survival rate and the increased progression and recurrence or metastatic rate. The high expression level of RAE1 was reported as an independent poor prognostic factor for colorectal cancer [13]. Moreover, the expression level of RAE1 was found to have the ability to accurately distinguish tumor tissues from normal tissues, and it could predict the 1-, 3-, and 5-year survival rates, which suggested that it could be used as a potential biomarker for the diagnosis and prognosis of HCC patients. However, the rate of RAE1 genetic alterations was only about 1.1% and the genetic alterations were not significantly associated with poor OS and DFS in HCC patients, although genetic mutations were closely associated with malignant tumors and a poor prognosis.

To explore the biological functions of RAE1, the LinkedOmics database was used to perform co-expression analysis and functional enrichment analysis in HCC samples. The results demonstrated that RAE1 co-expressed genes were significantly related to chromosome segregation and cell cycle checkpoints. A total of 5 common genes, including NUP205, NUP155, NUP214, NUP54, and NXF1 were screened through comparing RAE1-associated genes with RAE1-interacted genes. Importantly, these five genes were significantly positively correlated with RAE1. NPCs are composed of several copies of ∼ 30 different proteins called nucleoporins (Nups), playing key roles in cell division and mitotic checkpoint [35–37]. Moreover, several Nups have been reported to be associated with diverse types of cancer due to chromosomal translocations [38, 39]. A previous study demonstrated that RAE1 was a microtubule-associated protein and a mitotic checkpoint regulator [17]. These findings revealed that RAE1 could play a role in regulating cell cycle and chromosome segregation.

In conclusion, RAE1 may be a potential prognostic biomarker of a poor survival, and our findings may assist clinicians in the development of further efficacious therapeutic strategies for HCC (Additional file 1).

## Supplementary Information

**Additional file 1**. Clinical data of LIHC patients from TCGA.

## Data Availability

The datasets supporting the conclusions of this article are included within the article and its additional files.
